# Extreme Carcinoembryonic Antigen Elevation Revealing Clinically Silent Colonic Metastases From Gastric Adenocarcinoma: A Case Report

**DOI:** 10.7759/cureus.114667

**Published:** 2026-08-17

**Authors:** Ann Tanyous, Iten Tanyous, Alaa-Salah Eldin

**Affiliations:** 1 Research, Touro College of Osteopathic Medicine, Middletown, USA; 2 Department of Gastroenterology and Hepatology, Hudson Regional Hospital, Secaucus, USA

**Keywords:** cancer of unknown primary, carcinoembryonic antigen, colonic metastasis, esophagogastroduodenoscopy (egd), gastric adenocarcinoma, liquid biopsy, positron emission tomography, stomach neoplasms, virchow node, colonoscopy

## Abstract

Gastric adenocarcinoma rarely metastasizes to the colon, and extreme carcinoembryonic antigen (CEA) elevation exceeding 1,000 ng/mL occurs in approximately 1.3% of gastric cancers, where it is strongly associated with lymphatic dissemination and poorly differentiated histology. We report the case of a 47-year-old man with progressive constitutional symptoms in whom markedly elevated CEA prompted a comprehensive diagnostic workup, ultimately revealing stage IV gastric adenocarcinoma with clinically silent colonic metastases. Computed tomography demonstrated numerous pulmonary nodules, mediastinal and hilar lymphadenopathy, and a 3.5-cm periceliac mass with diffuse retroperitoneal and retrocrural adenopathy, without hepatic lesions or peritoneal implants. Initial CEA exceeded 600 ng/mL and subsequently rose to 1,170 ng/mL. Notably, endoscopy performed four years earlier had revealed only mild chronic erosive gastritis without *Helicobacter pylori* infection or intestinal metaplasia, and colonic biopsies were normal. Initial esophagogastroduodenoscopy during the current presentation showed gastric ulcers without a discrete mass, with nondiagnostic biopsies. Fine-needle aspiration of the Virchow node confirmed adenocarcinoma, but the primary site remained indeterminate. Positron emission tomography demonstrated hypermetabolic activity in the stomach (standardized uptake value 9) and left supraclavicular node (standardized uptake value 11), and liquid biopsy (Guardant360) confirmed an upper gastrointestinal primary. Follow-up colonoscopy revealed a friable submucosal mass in the transverse colon and multiple hemorrhagic ulcerated lesions in the descending colon, confirmed as metastatic gastric adenocarcinoma. A systematic review identified only 26 reported cases of colonic metastasis from gastric cancer. This case demonstrates that extreme CEA elevation (>1,000 ng/mL) should prompt comprehensive metabolic imaging and repeat targeted endoscopy even when initial biopsies are negative, and underscores the aggressive natural history of gastric adenocarcinoma, while acknowledging that an apparently benign prior endoscopy may reflect unsampled early disease rather than true de novo progression within years.

## Introduction

Gastric adenocarcinoma is the fifth most common cancer and the fifth leading cause of cancer-related mortality worldwide, with approximately one million new cases and 760,000 deaths annually [1-2]. Metastatic stage IV disease most commonly involves the liver (26-48%), peritoneum (32-46%), lung (15%), and distant lymph nodes (11-20%), with a five-year survival rate of less than 10% [2-3]. Colonic metastasis from gastric cancer is exceedingly rare. A systematic review by Fretwell et al. identified only 26 cases in the literature, with wide variation in presentation and management [4]. These metastases typically occur via hematogenous spread and present with characteristic submucosal thickening, often mimicking primary colorectal neoplasms [4-5].

Carcinoembryonic antigen (CEA) is a well-established tumor marker in gastrointestinal malignancies, although its sensitivity in gastric cancer is lower than in colorectal cancer. Elevated levels (>5 ng/mL) have been reported in 40-50% of patients with metastatic gastric carcinoma [6]. A meta-analysis of 14,651 patients confirmed that elevated pretreatment CEA is an independent prognostic factor in gastric cancer, associated with a nearly doubled risk of mortality (hazard ratio (HR) 1.716, 95% confidence interval (CI) 1.594-1.848) [7]. Extreme CEA elevation exceeding 1,000 ng/mL is exceptionally rare, occurring in only 1.3% of gastric cancers, and has been associated with signet ring cell or poorly differentiated histology and extensive lymphatic dissemination rather than hepatic metastasis [8].

The left supraclavicular lymph node, known as Virchow's node, has been recognized since 1848 as a sentinel indicator of intra-abdominal malignancy. Cervin et al. demonstrated that abdominal and pelvic malignancies preferentially metastasize to the left supraclavicular lymph node via the thoracic duct, and fine-needle aspiration of this site is an excellent initial diagnostic procedure [9-10]. In the setting of cancer of unknown primary (CUP), comprehensive genomic profiling through liquid biopsy has emerged as a valuable diagnostic tool, with circulating tumor DNA (ctDNA) analysis capable of identifying actionable mutations in 80% of CUP patients [11]. The CUPISCO trial, the largest interventional randomized trial in CUP to date, demonstrated that molecularly guided therapy significantly improved progression-free survival compared with standard platinum-based chemotherapy [12].

We present the case of a 47-year-old man with extreme CEA elevation who was found to have stage IV gastric adenocarcinoma with rare colonic metastases, diagnosed through a multimodal approach incorporating Virchow node biopsy, positron emission tomography (PET) imaging, liquid biopsy, and repeat targeted endoscopy. Notably, endoscopy four years earlier demonstrated apparently benign gastric mucosa without documented precancerous changes; however, the possibility of occult disease that was not detected or sampled at that examination cannot be excluded.

## Case presentation

A 47-year-old man presented to the emergency department with progressive back pain of approximately two months' duration, accompanied by anorexia and significant weight loss. His past medical history was notable for a prior right nephrectomy and diverticulosis. Home medications included analgesics for pain management. He reported no known drug allergies.

Four years prior to presentation, the patient had undergone esophagogastroduodenoscopy (EGD) and colonoscopy for evaluation of abdominal pain, nausea, vomiting, gastrointestinal bleeding, and diarrhea. Pathology from that evaluation demonstrated mild chronic erosive gastritis negative for *Helicobacter pylori *and intestinal metaplasia, and colonic biopsies showed normal mucosa.

On physical examination, the patient appeared his stated age and was in moderate distress. A palpable left supraclavicular lymph node (Virchow node) was identified. Head, eyes, ears, nose, and throat examination was unremarkable without icterus. Neck examination revealed no jugular venous distension or additional masses. Cardiovascular examination demonstrated regular rate and rhythm. Lungs were clear to auscultation bilaterally. The abdomen was soft, nontender, and nondistended. Extremities showed no clubbing or cyanosis. Skin was without jaundice.

Computed tomography (CT) of the chest, abdomen, and pelvis revealed numerous pulmonary nodules, mediastinal and hilar lymphadenopathy, diffuse retroperitoneal and retrocrural adenopathy, and a periceliac mass measuring 3.5 cm, without hepatic lesions, peritoneal implants, or ascites. 

Initial serum CEA was markedly elevated at greater than 600 ng/mL, with serial measurement during the same admission subsequently rising to 1,170 ng/mL. Ultrasound-guided fine-needle aspiration of the left supraclavicular lymph node was performed by interventional radiology; however, the initial sample was insufficient for diagnosis. Subsequent excisional biopsy of the Virchow node confirmed adenocarcinoma of indeterminate primary site. 

**Figure 1:**
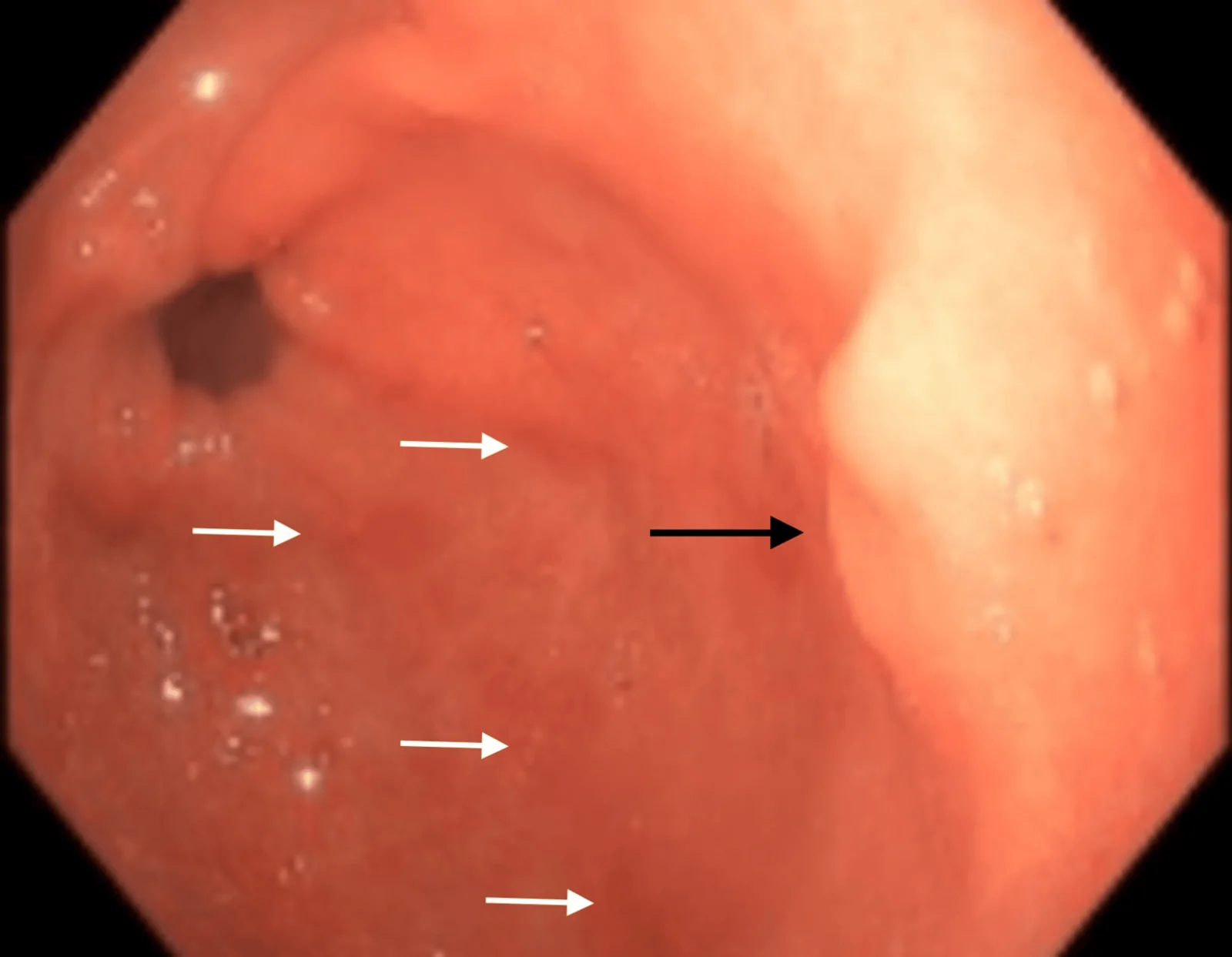
Esophagogastroduodenoscopy demonstrating infiltrative gastroesophageal junction abnormality suspicious for occult gastric adenocarcinoma. Endoscopic image of the gastroesophageal junction showing diffuse erythematous and infiltrative mucosal changes. White arrows indicate areas of mucosal irregularity and nodularity with subtle submucosal prominence. The black arrow indicates focal wall thickening/infiltrative mucosal distortion concerning for malignant involvement despite the absence of a discrete exophytic mass lesion.

EGD revealed diffuse gastric mucosal abnormalities without a discrete exophytic mass (Figure 1). Findings included erythematous and edematous gastric folds, mucosal congestion, and multiple shallow ulcerative and erosive lesions involving the gastric body and antrum. The infiltrative appearance raised concern for occult gastric malignancy despite initially nondiagnostic biopsies.

PET/CT demonstrated hypermetabolic activity in the pulmonary nodules, diffuse adenopathy (left supraclavicular node SUV 11), and focal gastric uptake (SUV 9). Based on the PET findings, markedly elevated CEA, and distribution of metastatic disease, a gastric primary was considered most likely.

Liquid biopsy was performed using the Guardant360 platform, including cancer signal of origin analysis, next-generation sequencing (NGS), and ctDNA evaluation. Results were most consistent with an upper gastrointestinal primary. 

The patient's clinical course was complicated by intractable abdominal pain and hematemesis requiring emergency department presentation. Pain management was initiated with intravenous hydromorphone and subsequently escalated to a fentanyl transdermal patch (titrated to 75 mcg/hr) with hydromorphone for breakthrough pain. Nausea and vomiting were managed with ondansetron and intravenous hydration. The patient reported anorexia with continued weight loss but denied fever, chills, shortness of breath, chest pain, headache, or visual changes.

A port was placed, and the patient was initiated on FOLFOX (leucovorin, fluorouracil, and oxaliplatin) chemotherapy. Following cycle one, the patient was readmitted for persistent nausea, vomiting, and pain despite antiemetic therapy and intravenous fluids. He reported significant fatigue and weakness. He was discharged with plans to continue cycle two as an outpatient, with ongoing monitoring of CEA and ctDNA.

**Figure 2:**
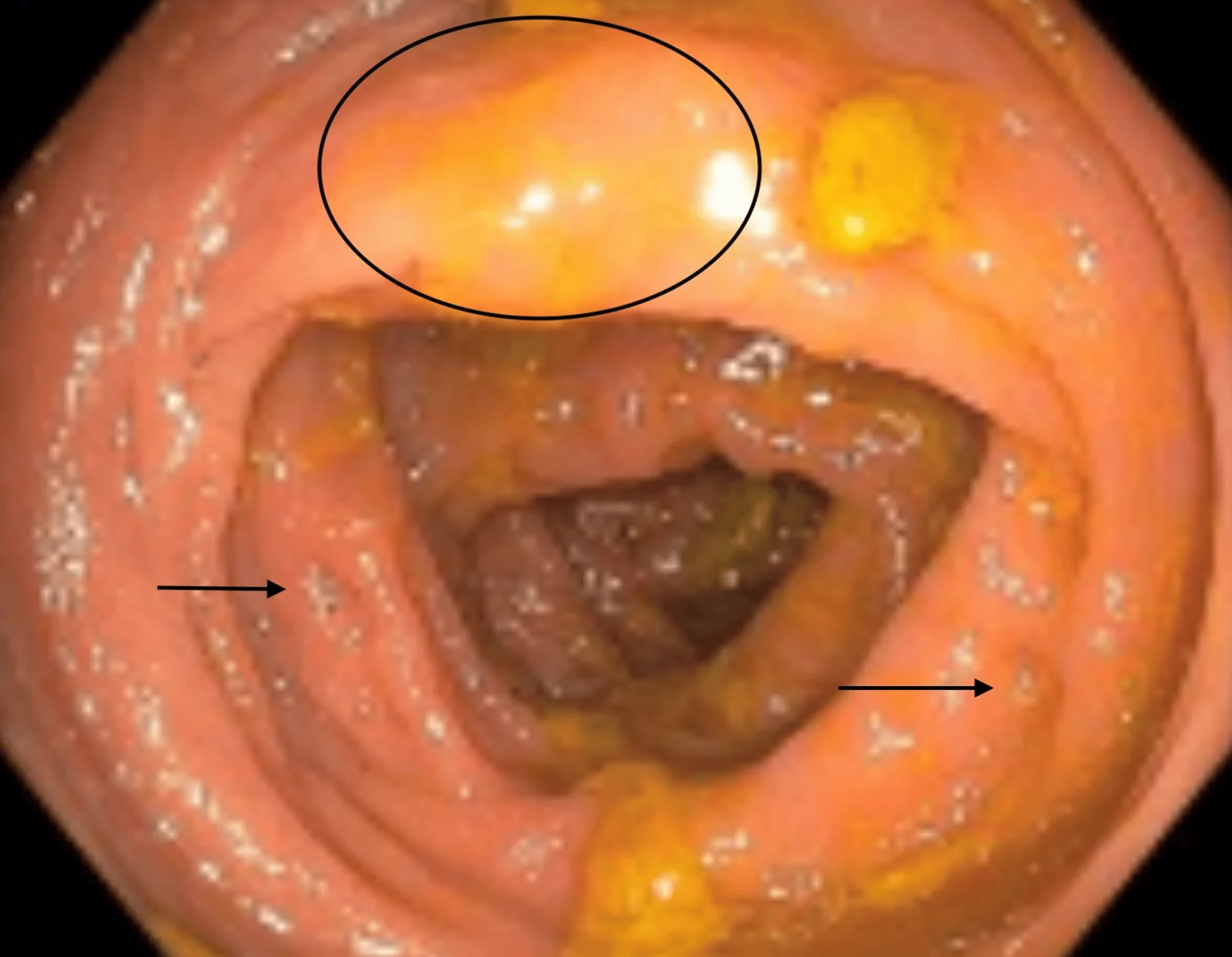
Colonoscopy demonstrating metastatic colonic involvement Colonoscopy revealed friable submucosal infiltrative lesions within the transverse colon (black arrows), resulting in partial luminal narrowing and distortion of the normal colonic architecture. The affected segment demonstrated marked edema, mucosal friability, and circumferential infiltrative thickening with a submucosal mass-like appearance (black circle). These endoscopic findings were highly suspicious for metastatic adenocarcinoma involving the colon rather than a primary colorectal malignancy.

**Figure 3:**
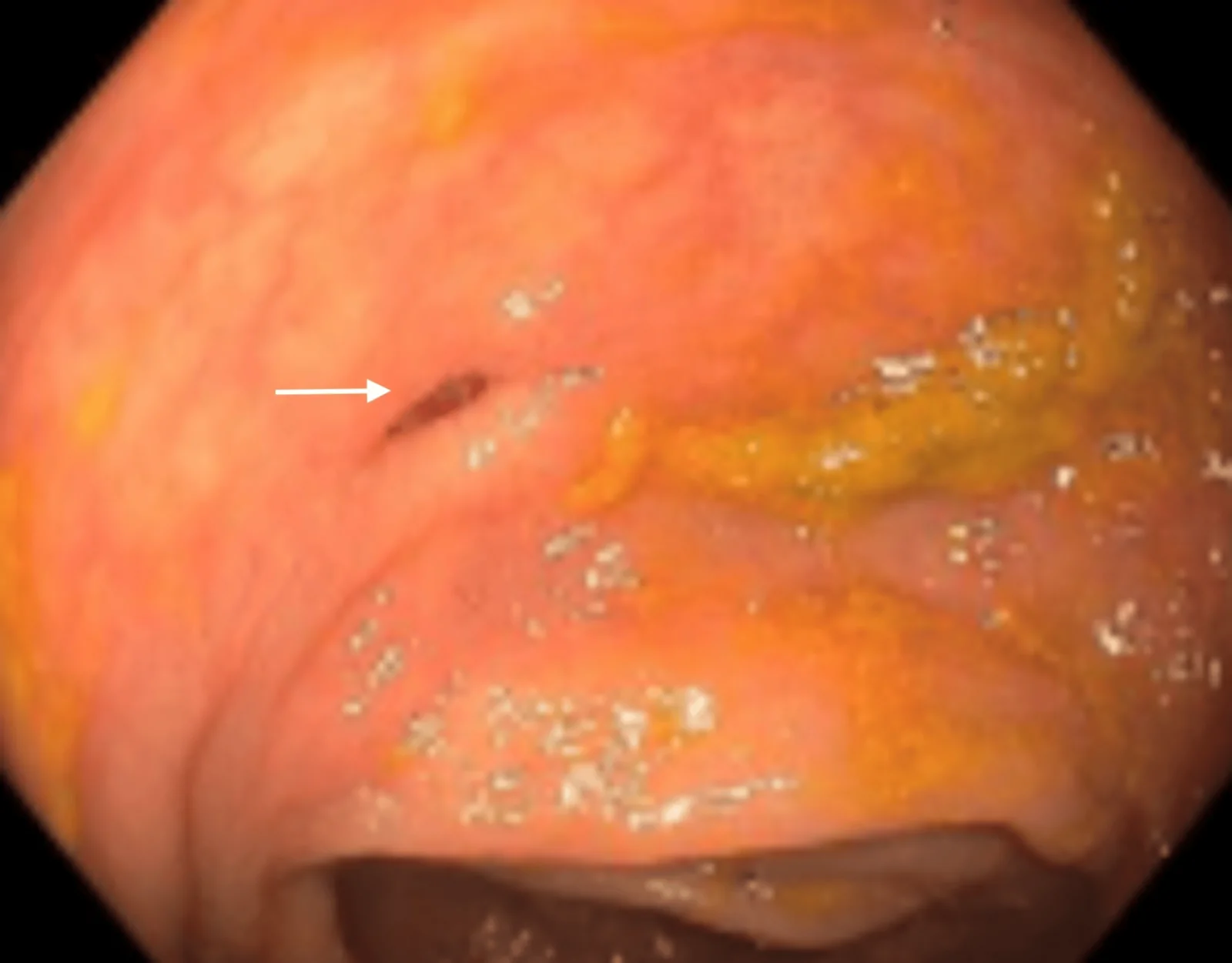
Colonoscopy findings in the descending colon Colonoscopy of the descending colon demonstrated a focal hemorrhagic ulcerative lesion (white arrow) with surrounding mucosal edema and irregularity. The ulcer base contained adherent fibrinous exudate and necrotic debris, producing an infiltrative appearance suspicious for metastatic involvement. These findings were consistent with metastatic deposits from gastric adenocarcinoma involving the colonic mucosa rather than a primary colonic ulcerative lesion.

Follow-up colonoscopy demonstrated a friable submucosal infiltrative mass within the transverse colon causing partial luminal narrowing and distortion of the normal colonic architecture, with associated edema, mucosal friability, and circumferential wall thickening concerning for metastatic adenocarcinoma rather than a primary colorectal neoplasm (Figure 2). In the descending colon, an irregular hemorrhagic ulcerative lesion with adherent fibrinous exudate and necrotic debris was identified, consistent with metastatic involvement of the colonic mucosa from gastric adenocarcinoma (Figure 3). Histopathologic examination confirmed metastatic gastric adenocarcinoma involving the colon.

## Discussion

This case illustrates several noteworthy clinical features: extreme CEA elevation in gastric cancer, apparent rapid progression from a prior benign endoscopy to stage IV disease, the rare finding of colonic metastases from a gastric primary, and the diagnostic utility of liquid biopsy in cancer of unknown primary.

The degree of CEA elevation in this patient (>1,170 ng/mL) is exceptional. In a study of 310 gastric carcinoma cases, Horie et al. found that only four patients (1.3%) demonstrated CEA levels exceeding 1,000 ng/mL, and these were associated with signet ring cell or poorly differentiated histology with extensive lymphatic rather than hepatic dissemination [8]. Multivariate regression analysis in that study identified lymph node metastasis as the only independent factor associated with marked CEA elevation, and patients with markedly elevated CEA had significantly higher rates of peritoneal metastasis (P < 0.0001) and lymph node metastasis (P < 0.05) compared with those with moderate elevations [8]. The extreme CEA elevation in our patient, in the absence of hepatic metastases but with diffuse lymphadenopathy, is consistent with this pattern. A meta-analysis of 14,651 patients confirmed that elevated pretreatment CEA is an independent prognostic factor in gastric cancer, associated with significantly poorer overall survival (HR 1.716, 95% CI 1.594-1.848), disease-specific survival (HR 1.940, 95% CI 1.563-2.408), and disease-free survival (HR 2.275, 95% CI 1.836-2.818) [7]. Tachibana et al. further demonstrated that preoperative serum CEA was an independent prognostic factor by multivariate analysis (P = 0.0003), with five-year disease-specific survival rates of 31.7% in CEA-positive versus 77.3% in CEA-negative patients [13]. The rapid rise from greater than 600 to 1,170 ng/mL during hospitalization reflects the aggressive tumor biology and high disease burden.

The apparent progression from benign gastric mucosa to advanced malignancy within approximately four years is striking, if the prior endoscopy is taken as a true negative. The Correa cascade describes the stepwise progression from normal gastric mucosa through chronic gastritis, atrophic gastritis, intestinal metaplasia, dysplasia, and ultimately adenocarcinoma, a process typically driven by chronic *H. pylori *infection and evolving over two to three decades or more [1,14]. In this patient, endoscopy four years prior demonstrated only mild chronic erosive gastritis without *H. pylori* infection or intestinal metaplasia, and colonic biopsies were entirely normal. The compressed timeline raises the possibility of a non-intestinal pathway of carcinogenesis, potentially consistent with diffuse-type gastric cancer, which does not necessarily follow the traditional Correa cascade and may arise through distinct molecular mechanisms including CDH1 mutations [1]. Importantly, chronic inflammation is not necessary for diffuse-type cancers, indicating distinct mechanisms in *H. pylori*-induced malignant disease [1]. Alternatively, and importantly, the prior endoscopy may have represented a false-negative examination rather than true benign mucosa. Early gastric neoplasia and diffuse-type infiltrative cancers are notoriously difficult to capture on random biopsies, and a submucosal infiltrative lesion may evade detection on both endoscopic inspection and mucosal sampling. Supporting the frequency of missed precursor disease, a recent study found that only 35.6% of gastric adenocarcinoma resection specimens had precursor lesions, and among those, only 26.2% had undergone prior EGD, with only 36.4% having previously documented precursor lesions. Thus, an occult early or diffuse-type lesion present but unsampled four years earlier is at least as plausible as de novo rapid malignant transformation [15].

Colonic metastases from gastric cancer are extremely rare. A systematic review by Fretwell et al. identified only 26 cases in the literature, noting wide variation in presentation and practice, with cases tending to occur in patients with poor histopathological features [4]. Su et al. reported that colonic metastases from gastric cancer frequently exhibit characteristic submucosal swelling and segmental bowel wall thickening on colonoscopy [5]. Jang et al. described the most common CT finding as target-like concentric bowel wall thickening involving multiple long segments, with the ascending colon (52%) and rectum (48%) being the most frequently involved sites, predominantly in patients with linitis plastica and poorly differentiated histology [16]. Kohno et al. reported a case of colorectal metastasis diagnosed eight years and 10 months after gastrectomy, emphasizing that colonoscopic findings provide useful diagnostic information and that biopsy pathology may not always reveal malignancy, sometimes necessitating surgical resection for definitive diagnosis [17]. In our patient, the colonoscopic findings of a friable submucosal mass in the transverse colon and hemorrhagic ulcerated lesions in the descending colon are consistent with these descriptions and were confirmed histopathologically as metastatic gastric adenocarcinoma.

The diagnostic approach in this case highlights the evolving role of liquid biopsy in cancer of unknown primary. The National Comprehensive Cancer Network (NCCN) guidelines for occult primary recommend biomarker testing using NGS on tumor tissue and/or cell-free DNA testing [18]. The CUPISCO trial demonstrated that molecularly guided therapy significantly improved progression-free survival compared with standard platinum-based chemotherapy in unfavorable CUP, supporting the use of comprehensive genomic profiling in the diagnostic workup [12]. Kato et al. demonstrated that ctDNA evaluation is feasible in CUP, with 80% of patients exhibiting ctDNA alterations and 99.7% harboring potentially targetable alterations [11]. In our case, liquid biopsy was instrumental in confirming an upper gastrointestinal primary when tissue biopsies were initially nondiagnostic, supporting the growing evidence for incorporating ctDNA analysis into the diagnostic workup of CUP.

The presence of a Virchow node provided the initial diagnostic clue. Adler et al. reviewed the clinical and radiologic significance of Virchow's node, confirming that identification of a pathologically enlarged left supraclavicular node raises suspicion for abdominopelvic malignancy and provides a safe and accessible target for biopsy [10]. Cervin et al. demonstrated that abdominal and pelvic malignancies preferentially metastasize to the left supraclavicular lymph node via the thoracic duct [9]. In our patient, while the initial fine-needle aspiration yielded insufficient tissue, subsequent excisional biopsy confirmed adenocarcinoma, underscoring the importance of pursuing adequate tissue sampling when clinical suspicion is high.

Several limitations should be acknowledged. The histologic subtype of the gastric adenocarcinoma (intestinal vs. diffuse) was not specified in the available records. Details regarding HER2, PD-L1, CLDN18.2, and microsatellite instability/mismatch repair status were pending at the time of this report and would be critical for optimizing treatment selection. The mechanism of colonic metastasis (hematogenous vs. direct extension vs. peritoneal seeding) could not be definitively established, though the submucosal pattern of involvement favors hematogenous spread. Additionally, the apparent four-year progression is inferred from a single prior endoscopy; a false-negative examination or biopsy sampling error cannot be excluded, and early or diffuse-type neoplastic changes may have been present but unsampled at that time. The reported timeline should therefore be interpreted with this caveat.

## Conclusions

This case illustrates the aggressive natural history of gastric adenocarcinoma, with an apparent progression from a prior benign endoscopy to stage IV disease with rare colonic metastases within approximately four years - recognizing that unsampled early disease at the prior examination cannot be excluded. The extreme CEA elevation (>1,170 ng/mL) served as a critical diagnostic signal that prompted comprehensive metabolic imaging and repeat targeted endoscopy, ultimately revealing clinically silent colonic metastatic deposits. Three lessons emerge for clinicians: extreme CEA elevation in the absence of hepatic metastases should raise suspicion for extensive lymphatic dissemination and unusual metastatic patterns, including rare sites such as the colon; liquid biopsy represents a valuable adjunct in the diagnostic workup when tissue biopsies are nondiagnostic, particularly in the setting of cancer of unknown primary; and clinicians should maintain a high index of suspicion for colonic metastases in patients with advanced gastric cancer, as these rare lesions may be amenable to targeted endoscopic detection.
